# The genetics of occupational asthma development among workers exposed to diisocyanates: A systematic literature review with meta-analysis

**DOI:** 10.3389/fgene.2022.944197

**Published:** 2022-10-06

**Authors:** Laura J. Word, Emily P. McAden, Charles Poole, Leena A. Nylander-French

**Affiliations:** ^1^ Environmental Sciences and Engineering, University of North Carolina at Chapel Hill, Chapel Hill, NC, United States; ^2^ Epidemiology, University of North Carolina at Chapel Hilll, Chapel Hill, NC, United States

**Keywords:** systematic literature review, meta-analysis, genetics, single nucleotide polymorphisms (SNPs), inflammation, diisocyanate asthma (DA), bioinformatics, suscepibility genes

## Abstract

Diisocyanates are widely used compounds that pose a safety concern for workers in occupations within the spray-paint, spray-foam insulation, and furniture varnish industries. Epidemiological studies show that only a subset of workers exposed to diisocyanates develop diisocyanate-induced occupational asthma (diisocyanate asthma, DA), indicating that genetic susceptibility may play a role. The purpose of this systematic literature review was to compile and meta-analyze the reported data on genetic susceptibility markers for DA. Three databases (Embase, Pubmed, and Scopus) were searched and 169 non-duplicate publications were identified, of which 22 relevant occupational studies were included in this review. Researchers reported prevalence odds ratios (PORs) for 943 comparisons in 82 different genes/serotypes. Protein network functions for the DA-associated genes from this review include: antigen processing, lymphocyte activation, cytokine production regulation, and response to oxidative stress. Meta-analysis of comparisons between workers with DA and controls was conducted for 23 genetic markers within: CTNNA3, *GSTM1, GSTP1, GSTT1, HLA-C, HLA-DQB1, HLA-DR1, HLA-DR3, HLA-DR4, HLA-DR7,* and *HLA-DR8*. These genes code for proteins that are involved in cell-cell adhesions (*CTNNA3*), glutathione conjugation for xenobiotic metabolism (*GST* gene family), and immune system response (*HLA* gene family). The most compelling pooled PORs were for two studies on CTNNA3 (increased DA risk: rs10762058 GG, rs7088181 GG, rs4378283 TT; PORs 4.38–4.97) and three studies on HLA-DR1 (decreased DA risk, POR 0.24). Bioinformatics of the predicted protein pathways for DA shows overlap with biomarker-associated pathways in workers before development of asthma, suggesting overlap in toxicokinetic and toxicodynamic pathways of diisocyanates. The control groups were also compared against each other and differences were negligible. Suggestions for improving future research are also presented. Of the highest importance, the literature was found to be profoundly publication-biased, in which researchers need to report the data for all studied markers regardless of the statistical significance level. We demonstrate the utility of evaluating the overlap in predicted protein pathway functions for identifying more consistency across the reported literature including for asthma research, biomarker research, and *in vitro* studies. This will serve as an important resource for researchers to use when generating new hypothesis-driven research about diisocyanate toxicology.

## Introduction

Diisocyanates are a commonly used class of chemicals that are reacted with polyols to produce polyurethane and are one of the most common causes of occupational asthma (Malo and Chan-Yeung 2009; Dao and Bernstein 2018). The most abundantly produced diisocyanates are toluene diisocyanate (TDI), methylene diphenyl diisocyanate (MDI), and 1,6-hexamethylene diisocyanate (HDI) (Randall and Lee, 2002). The prevalence of isocyanate-induced asthma has been difficult to estimate; estimates within various industries range from less than 1% to more than 30% (Lockey et al., 2015). Part of the challenge for estimating the prevalence of diisocyanate asthma (DA) is the healthy-worker effect (Chowdhury et al., 2017), in which the worker population is healthier than the general population because a worker who develops a health problem often leaves the job. And even though the increase in precautions to reduce exposure to isocyanates has helped to decrease the percentage of workers with health complaints, the increase in yearly production of isocyanates will still result in an increase in the global burden of disease caused by isocyanates (Verschoor and Verschoor 2014).

Every case of DA is very costly. An assessment performed in the United Kingdom (UK) for occupational asthma cases from 2003 showed that occupational isocyanate-asthma cases result in millions of Great Britain Pounds (GBP) in direct and indirect costs from healthcare and lost work and pay (Ayres et al., 2011). Over their lifetimes, 108 workers who developed isocyanate-asthma in the UK in 2003 were estimated to cost individuals, employers, and taxpayers between £13,688,000 to £14,729,000 in 2004 currency rates (Ayres et al., 2011). Additionally, DA can have a poor prognosis. In a two-year study, 50% of patients continued to display asthma symptoms at the end of the study (Park and Nahm 1997) and the best medical course of action is still to remove the worker from further exposure to diisocyanates (Park and Nahm 1997; Verschoor and Verschoor 2014). Therefore, it is important to better understand diisocyanate sensitization and factors that impact worker susceptibility differences. Because only a subset of workers develops DA, there is thought to be an underlying genetic component that contributes to inter-individual susceptibility to DA.

In order to investigate genetic markers of susceptibility, multiple research groups have primarily conducted candidate gene studies, and a few have conducted genome-wide association studies (GWAS). An early investigation in the field identified that slow acetylators were disproportionately present among ten of eleven (91%) DA cases (Berode 1991; Berode and Savolainen 1993). The study was later replicated in a group of 47 DA cases from Switzerland, of whom 33 (70%) had a slow *N*-acetyltransferase (*NAT*) genotype (Berode et al., 2005). Other early investigations studied associations between DA and human leukocyte antigen (*HLA*) serotypes, which are involved in antigen presentation (Bignon et al., 1994; Fabbri et al., 1995; Balboni et al., 1996; Bernstein et al., 1997; Rihs et al., 1997), and many investigators found significant associations.

The objective for this systematic literature review with meta-analysis was to identify all studies in which associations between genetic markers and the development of DA were investigated in diisocyanate-exposed worker populations (see Table 1). Gaining a better understanding of the genetic markers that increase susceptibility to DA has the potential to help identify important genetic pathways that are involved in the mechanisms for the development of DA. The DA-related gene results were also compared to the genetic pathways that are associated with differences in diisocyanate biomarker levels following occupational exposure. Put together, these gene lists can help researchers to better understand the cell signaling pathways that impact the toxicokinetics and toxicodynamics of diisocyanates. This analysis on DA can improve understanding of this complex disease and its gene-disease associations, which can help to determine the mechanism of isocyanate sensitization and identify better protections for workers.

**TABLE 1:** The Preferred Reporting Items for Systematic reviews and Meta-Analyses (PRISMA) recommended description of the participants, interventions, comparisons, outcomes, and study design (PICOS) targeted in this systematic review.

PICOS category	Description
Participants	Human adult workers
Interventions/exposure	Occupational diisocyanate exposure
Comparison	Individuals without DA
Outcome	Occupational DA
Study designs	All study designs (included case-control candidate-gene studies and GWAS)

DA, diisocyanate-induced asthma; GWAS, genome-wide association study.

## Methods

### Literature search

Librarians Mary White and Jennifer Walker at the University of North Carolina at Chapel Hill were consulted for guidance to conduct the literature search on human genetic susceptibility markers for the development of DA from occupational isocyanate exposure. The electronic databases Embase, Pubmed, and Scopus were queried on 27 February 2019 (without publication date restrictions on the results) and again on 30 January 2020. Synonyms of genetics, diisocyanates, and occupational asthma were used as the search terms (see Supplementary Excel File). Medical Subject Headings (MeSH) terms were used in Pubmed and were adapted for Embase and Scopus. Duplicate publications were removed and then two independent researchers screened the titles and abstracts for relevance (Laura Word and Emily McAden), followed by full text review to evaluate the studies for inclusion (i.e., whether they met all of the inclusion criteria and none of the exclusion criteria). A third scientist settled any sorting differences that arose (Leena Nylander-French). Zotero reference manager (https://www.zotero.org; Mueen Ahmed and Dhubaib. 2011) was used for management of the literature during the sorting process.

### Inclusion and exclusion criteria

All original research study designs that reported associations between genetic markers and susceptibility for development of DA were included, e.g., case-control candidate gene studies and GWAS. Letters to the editor, abstracts, reviews, and books were excluded. Off-topic studies, murine model *in vivo* studies, and *in vitro* studies were also excluded. The remaining papers were independently screened for details about the medical DA diagnosis and for descriptions of genetic susceptibility polymorphisms for DA as compared to at least one control group without DA. The most common control groups consisted of exposed workers without symptoms (asymptomatic workers, AW), exposed workers with asthma symptoms for whom DA was ruled out via a negative specific inhalation challenge (DA negative, DA-), and non-exposed healthy individuals (normal controls, NC). All data for DA-, AW, and NC were extracted in the systematic review.

### Inclusion criteria

1. Peer-reviewed research
2. Subjects are adult workers who were medically diagnosed with occupational DA
3. Includes and describes at least one control group without DA
4. Genetic susceptibility markers for DA are described compared to the control group(s)

### Exclusion criteria

1. Animal model study
2. Cell culture study

### Assessment of quality and bias

Individual studies were assessed for their quality using a list of questions we generated that are particularly relevant to these studies. We used the Newcastle-Ottawa scale for case-control studies as a starting point to generate the list of study characteristics that we evaluated (Wells et al., 2014). For each study we evaluated: the rigor of the method of the determination of DA cases, whether Hardy-Weinberg Equilibrium (HWE) was evaluated in controls and whether it indicated evidence of population stratification, whether population stratification was assessed, and whether there was inclusion of potential covariates for DA such as smoking (Ucgun et al., 1998). Quality assessment was used to help assess bias potential and to make sure important study characteristics were considered in our analysis. The small number of studies per genetic marker prevented the drawing and analysis of funnel plots.

### Data extraction

The principal measures collected were genetic polymorphisms and their associated genes and prevalence odds ratios (POR) for susceptibility to developing occupational DA. Information was also collected on: study design, study populations’ demographic characteristics, number of participants, type and duration of isocyanate exposure, health and exposure status of the controls, testing for HWE in controls, information on whether corrections for multiple comparisons were made, and which potential confounding factors were adjusted for in logistic regression models. All extracted information is in the Supplementary Excel File.

### Contacting authors

All research groups that had reported findings on the impact of genetics on the prevalence of DA in workers were contacted in an attempt to gather missing information that was not reported but no one was able to supply additional information on their studies. This was stated to be due to records disposition or the record keeper becoming unreachable after leaving the position.

### Bioinformatics

To learn more about which protein networks might be involved in the development of DA, an online tool called GeneMANIA (https://www.genemania.org; Warde-Farley et al., 2010) was used. GeneMANIA uses a label propagation algorithm to predict direct and indirect network interactions based off of validated unbiased protein-protein and protein-DNA interactions (Warde-Farley et al., 2010; Zuberi et al., 2013). We used GeneMANIA to identify the predicted protein network interactions for all genes that were reported by two or more studies to have DA-associated genetic markers. For this assessment, GeneMANIA was used to search for co-expression, co-localization, genetic interactions, pathways, physical interactions, predicted networks, and shared protein domains using the molecular function-based gene-ontology weighting setting. Predicted protein network functions for DA-associated genetic markers were also compared to predicted protein networks for DA-associated epigenetic markers as well as genetic and epigenetic markers associated with diisocyanate-biomarker variability.

### Meta-analysis

All genetic markers with reported results from two or more studies were meta-analyzed using the metan package (Harris et al., 2008) from Stata/SE 16.0 for Windows (StataCorp, 2019) to calculate pooled PORs and to make forest plots. Many researchers compared workers with DA to two different control groups. When multiple control groups were studied it was a combination of DA- and AW controls, or AW and NC controls. In other studies, only one control group, either AW or NC controls, were studied. For our main analysis evaluating genetic markers associated with differences in worker risk of developing DA, we used data for AW workers as the preferred control group, followed by NC. Therefore, DA-controls were not used in our main analyses (since AW worker data was always available when DA-controls were studied). This control group order preference is explained further in the discussion section.

The authors reported odds ratios that were for prevalence of DA amongst the people in their studies and thus are actually PORs. The POR assessments that we meta-analyzed included several types of comparisons. These included PORs for null *vs.* present for some genes, and the homozygous major allele *vs.* heterozygous and homozygous minor allele (dominant model), heterozygous genotype *vs.* homozygous major allele and homozygous minor allele, and homozygous minor allele *vs.* homozygous major allele and heterozygous genotype (recessive model) for single nucleotide polymorphisms (SNPs).

Meta-analyzed genetic markers were not categorized as being statistically significant due to the small number of studies and the need for further research before any conclusions are drawn about the associations between the genetic markers and DA. Instead, genetic markers were arranged by the precision of the estimates. Precision was determined by calculating ratios of the pooled upper 95% confidence interval (UCI) divided by the lower 95% confidence interval (LCI), UCI/LCI, in which ratios that are closer to 1 demonstrate that the gathered data is more precise. It is important to note that the strength of the genetic markers’ association with DA may change with further research.

### Sensitivity analysis

Sensitivity analysis was used to investigate whether the type of controls used for analysis altered the meta-analysis findings. Many of the studies had used two separate control groups, either a combination of DA- and AW or both AW and NC. In this situation, we had to decide which data to use in the main meta-analysis. We decided to use AW controls if available and NC data if AW controls were not a part of the study. Thus, sensitivity analysis was needed to evaluate the impact of that decision on the analysis results. The sensitivity analysis consisted of using biologically-relevant ranked lists of preference to decide which control group data to include in each analysis when multiple control groups were used in a single study. When authors reported PORs adjusted for potential confounders, we also ran separate analyses for each combination of control group rank ordering using the non-adjusted and the author-adjusted PORs when available. There were 23 genetic markers that were studied by two or more research groups and we were able to evaluate with meta-analysis and this sensitivity analysis. The rank ordering of which control group was used for the analyses were: (1) AW,NC, (no DA-) using unadjusted PORs [primary analysis], (2) AW,NC, (no DA-) using author adjusted PORs, (3) NC,AW, (no DA-) using unadjusted PORs, (4) NC,AW, (no DA-) using author adjusted PORs, (5) DA-,AW, NC using unadjusted PORs, (6) DA-,AW, NC using author adjusted ORs, and (7) combined control data when a study had more than one control group. The variables that the researchers adjusted for included: age, atopy, duration of exposure, sex, smoking status, and/or type of exposure. Another component of the sensitivity analysis included calculating PORs using data from the 1,000 Genomes project (n = 2,504) (Fairley et al., 2020) as an alternative normal control group for the four SNPs that were in the meta-analysis (list 8 in Supplementary File).

When a study included two control groups, the control groups were also compared directly to each other. Pooled ORs for DA- vs. AW and AW vs*.* NC controls were calculated across all markers in studies with two control groups in order to evaluate whether there were appreciable differences in the genetics between the control groups. This can provide more information on how using different control groups might impact results when studying the relationship between genetics and DA risk. For this evaluation, the ORs of controls with and without the genetic marker genotype were compared directly to each other for each reported genetic marker and then meta-analyzed. For example, for AW *vs.* NC, the OR for each genetic marker was calculated using the equation:

This review complies with the Preferred Reporting Items for Systematic reviews and Meta-Analyses (PRISMA) recommendations (Moher et al., 2009).

## Results

### Study selection

**FIGURE 1:**
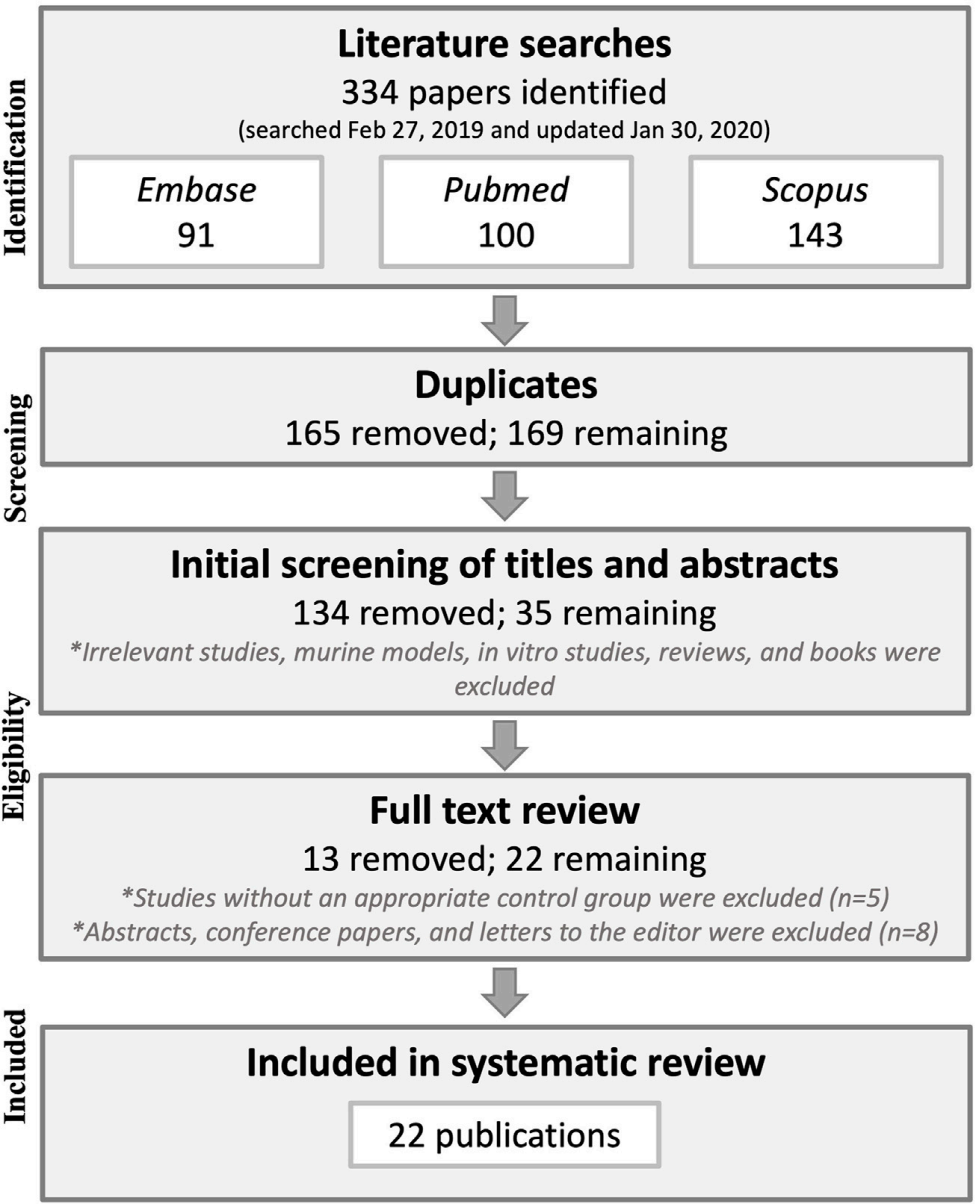
Preferred Reporting Items for Systematic reviews and Meta-Analyses (PRISMA) flowchart of the study selection methodology.

The database searches yielded 334 results, which were reduced to 169 after duplicate publications were removed. Title and abstract screening eliminated another 134 publications that did not pertain to the research question, were animal model or *in vitro* studies, and/or were not original research (letters to editors and reviews). The full text was then retrieved for the remaining 35 publications after which five publications lacking a control group and eight abstracts with later corresponding full-study publications were excluded, leaving 22 publications that were included in the systematic literature review. See Figure 1 for a flow chart of the methodology process for study selection.

No studies were excluded based on their method of DA diagnosis. Nearly all studies included workers who were diagnosed with DA based on a specific inhalation challenge (SIC) causing forced expiratory volume in one second (FEV1) to decrease by ≥ 20%. Three studies did not specify their definition for a positive test but did perform SIC testing (Bernstein et al., 1997; Piirilä et al., 2001; Wikman et al., 2002). Bernstein et al. (1997) tested some of the workers in the DA group with diisocyanates in the workplace instead of in a controlled laboratory setting. They also conducted lung function testing beforehand to establish increased clinical symptoms and peak flow variability changes at work versus away from work. Therefore, we considered this study to be methodologically acceptable for medically diagnosing DA and to be included in this literature review. Additionally, Ye et al. (2006) and Ye et al. (2010) included some workers who were diagnosed using interview, chest radiography, skin prick test with common inhalant allergens, and methacholine inhalation challenge (MIC), but most of their DA cases were diagnosed using SIC. Lastly, Bernstein et al. included 87 workers who had a positive SIC test and four workers who had peak expiratory flow monitoring during 2 weeks of work and 1–2 weeks off from work (Bernstein et al., 2018), and thus, this study was also deemed methodologically acceptable to be included in the literature review and meta-analysis.

### Systematic literature review findings

The 22 included publications reported data for 943 genetic marker comparisons (including haplotypes, serotypes, and different genotypes) within 82 different genes for DA versus DA-, AW, and/or NC controls. Information about the study designs and the full list of extracted PORs for all 22 publications are available in the Supplementary Excel File.

In the candidate-gene studies, genes whose tested genetic markers were reported to include significant associations within a study population were: HLA-DQA1, HLA-DQB1, HLA-DRB1, HLA-DR, GSTP1, GSTM1, NAT1, NAT2, HLA-A, IL4RA, HLA-C, HLA-Cw, SOD2 aka MnSOD, HOA1, SERPIN1, SERPINB3, EPHX1, HLA-B, HLA-E, HLA-DOA, HLA-DQA2, HLA-DPB1, MBL2, PTGS1, PTGS2, TGF-B, and TNF-a (Bignon et al., 1994; Balboni et al., 1996; Mapp et al., 2000; Mapp et al., 2002; Beghé et al., 2004; J Bernstein et al., 1997; Piirilä et al., 2001; Wikman et al., 2002; Kim et al., 2006; Ye et al., 2006; Choi et al., 2009; Ye et al., 2010; Kim et al., 2015; Bernstein et al., 2006; Yucesoy et al., 2012; Bernstein et al., 2013; Yucesoy et al., 2014; Yucesoy et al., 2015a; Yucesoy et al., 2016).

DA-prevalence associated genes from the GWAS studies were: CTNNA1, CTNNA3, GADL1, PFKFB3, PCNX, UGT2B4, CRTAC1, C11orf74 or RAG2, ALK, TUSC3, MLLT3 or SLC24A2, TRPM8, IBTK, KCNIP4, PDGFD, LHPP, DOCK2, PCTK2, ASTN2, C7orf13, SAMD12, IGF2BP1 or B4GALNT2, AHNAK, MAGI3 or LRIG2, ACMSD, CDH17, HERC2, NPAS3, ODZ3, PITPNC1, PRKCA, SLC6A12, TACR1, ZBTB16, ATF3, and FAM71A (Kim et al., 2009; Yucesoy et al., 2015b; Bernstein et al., 2018).

On the other hand, genes whose tested genetic markers were all insignificant within a given candidate-gene study population included: HLA-A, HLA-B, HLA-Cw, HLA-DRB1, HLA-DPB1, HLA-DQ, HLA-DQA1, HLA-DQB1, HLA-DR, GSTP1, GSTT1, IL13, CD14, TNF-a, NAT1, NAT2, NK2R, ADRB2, IL4RA, GSTM1, EPHX, ADAM33, ALOX5, GDF15, IL10, IL1a, IL1B, and IL1RN (Bignon et al., 1994; Balboni et al., 1996; Beghé et al., 2004; J Bernstein et al., 1997; Piirilä et al., 2001; Wikman et al., 2002; Kim et al., 2006; Ye et al., 2006; Choi et al., 2009; Ye et al., 2010; Bernstein et al., 2006; Yucesoy et al., 2012; Yucesoy et al., 2015a; Yucesoy et al., 2016). There are too many insignificant genetic markers from the GWAS studies to list.

Most of the time, research groups studied different genetic markers within the genes listed above. For a subset of those genes, in some studies genetic markers with significant associations were observed while in others no markers were identified within those genes that were associated with differences in the prevalence of DA: GSTP1, HLA-A, HLA-B, HLA-DPB1, HLA-DQA1, HLA-DQB1, HLA-DR, HLA-DRB1, NAT1, NAT2, and TNF-a.

### Bioinformatics

**FIGURE 2:**
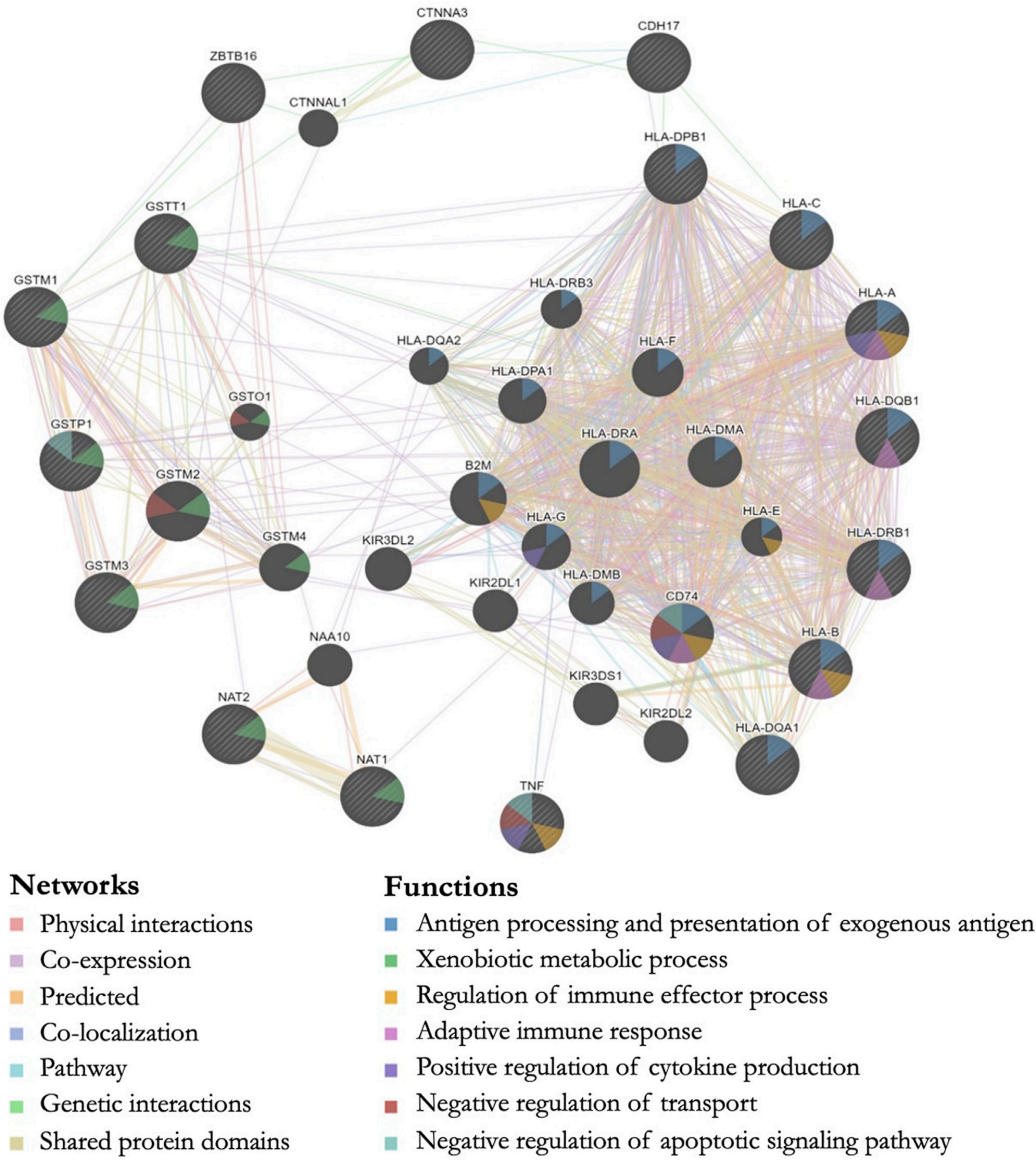
GeneMANIA output showing predicted protein networks between all genes with significant markers identified by multiple studies when the genetics of workers with diisocyanate-asthma (DA) was compared to asymptomatic workers (AW) or unexposed healthy normal controls (NC). Query genes have black circles with white-striped lines, the networks are shown with the colored lines between genes, and the shading on the circles shows the functions.

All genes with significant markers identified by multiple studies (Table 2) were input into GeneMANIA to evaluate overlapping protein networks for those genes. GeneMANIA shows that the DA-associated genes are often co-expressed and the protein network functions include immune system response, xenobiotic metabolism, transport regulation, and apoptosis regulation (Figure 2). When the protein network functions were compared with other genetic and epigenetic diisocyanate-related research for biomarker levels and epigenetic markers associated with DA, overlap was observed across different laboratories and studies (Table 3).

**TABLE 2:** Descriptions of genes that were included in the meta-analysis because two to three manuscripts reported markers within those genes with significant diisocyanate-asthma (DA) associations. Genetic markers that were significant only in combination with one or more other markers as a haplotype are not listed here. The genes in this table were reported to be associated with DA when genetics of workers with DA were compared to asymptomatic workers (AW) or unexposed healthy normal individuals (NC) as controls.

Gene	Full gene name	References	NCBI description
GSTM1	Glutathione-*S*-Transferase M1	Piirilä et al., (2001); Yucesoy et al., (2012) ^a^	Functions in the detoxification of electrophilic compounds, including. environmental toxins and products of oxidative stress, by conjugation with glutathione. [provided by RefSeq, July 2008]
GSTP1	Glutathione-*S*-Transferase P1	Mapp et al., (2002); Yucesoy et al., (2012)	This GST family member is ... thought to function in xenobiotic metabolism and play a role in susceptibility to cancer, and other diseases. [provided by RefSeq, July 2008]
HLA-B	Human Leukocyte Antigen B	Beghé et al., (2004); Yucesoy et al., (2014)	Class I molecules play a central role in the immune system by presenting peptides derived from the endoplasmic reticulum lumen. [provided by RefSeq, July 2008]
HLA-C	Human Leukocyte Antigen C	Beghé et al., (2004); Choi et al., (2009)	Class I molecules play a central role in the immune system by presenting peptides derived from endoplasmic reticulum lumen. [provided by RefSeq, August 2020]
HLA-DPB1	Human Leukocyte Antigen DPB1	Bignon et al., (1994); Yucesoy et al., (2014)	[HLA-DPB] plays a central role in the immune system by presenting peptides derived from extracellular proteins. [provided by RefSeq, July 2008]
HLA-DQA1	Human Leukocyte Antigen DQA1	Bignon et al., (1994); Balboni et al., (1996); Bernstein et al., (1997) ^b^; Mapp et al., (2000)	[HLA-DQA1] plays a central role in the immune system by presenting peptides derived from extracellular proteins. [provided by RefSeq, July 2008]
HLA-DQB1	Human Leukocyte Antigen DQB1	Bignon et al., (1994); Balboni, (1996); Bernstein et al., (1997) ^b^; Mapp et al., (2000); Choi et al., (2009)	HLA-DQB1 … is expressed in antigen presenting cells (APC: B lymphocytes, dendritic cells, macrophages). [provided by RefSeq, September 2011]
HLA-DRB1	Human Leukocyte Antigen DRB1	Bignon et al., (1994); Mapp et al., (2000) ^a^	HLA-DRB1 ... plays a central role in the immune system by presenting peptides derived from extracellular proteins. Hundreds of DRB1 alleles have been described and some alleles have increased frequencies associated with certain diseases or conditions. [provided by RefSeq, July 2020]
NAT1	*N*-acetyltransferase 1	Wikman et al., (2002); Yucesoy et al., (2015a) ^a^	This enzyme helps metabolize drugs and other xenobiotics, and functions in folate catabolism. [provided by RefSeq, August 2011]
NAT2	*N*-acetyltransferase 2	Wikman et al., (2002); Yucesoy et al., (2015a)	This gene encodes an enzyme that functions to both activate and deactivate arylamine and hydrazine drugs and carcinogens. Polymorphisms in this gene are also associated with ... drug toxicity. [provided by RefSeq, September 2019]
TNF-α	Tumor Necrosis Factor α	Beghé et al., (2004); Yucesoy et al., (2016) ^a^	This gene encodes a multifunctional proinflammatory cytokine. ... This cytokine is involved in the regulation of ... cell proliferation, differentiation, apoptosis, lipid metabolism, and coagulation. Knockout studies in mice also suggested the neuroprotective function of this cytokine. [provided by RefSeq, August 2020]

a only significant in logistic regression with other genetic markers included in the model.

b studied HLA-DQ, which overlaps with HLA-DQA1 and HLA-DQB1.

**TABLE 3:** Overlap between DA prevalence predicted protein network functions from this literature review and other diisocyanate research including exposure biomarker research, epigenetics of DA research, and *in vitro* studies. Genes with associations within the studies in the columns are grouped by protein function and show overlapping themes for the types of protein functions that are associated with diisocyanate studies.

Protein function	Genetics of DA (papers from this systematic review)	Genetics of TDI biomarker levels of TDA Broberg et al., (2010); Broström et al., (2018)	Genetics of HDI biomarker levels of HDA and TAHI Taylor et al., (2020)	Epigenetics of HDI biomarker levels of HDA and TAHI Taylor et al., (2021)	Epigenetics of DA Ouyang et al., (2013)	Miscellaneous diisocyanate exposure studies
Cell-adhesions	ATF3, CDH17, CTNNA3, ODZ3			KRT6A, NOSIP		
Immune system function and/or inflammatory response	DOCK2, GSTP1, HERC2, HLA gene family, MBL2, PTGS2 (aka COX-2), TACR1, TNF-α	P2PRX7	DGKB, GRK5	FZD9, LEPR	IFN-γ, IL-4	IFN-γ producing CD8^+^ T cells were detected in the bronchial mucosa of TDI-asthma patients Maestrelli et al., (1994)
*TGF-β pathway*	ALK, TGF-β		ACVR1, ACVR1C, GDNF, BMPR1B			
Xenobiotic metabolism	GSTM1, GSTP1, NAT1, NAT2	GSTP1				
Cell migration	ALK1, DOCK2		ETV1, GRK5, SALL1	KRT6A, FUZ		
Apoptosis regulation	LHPP		GDNF	MAPK10		MAPKs were found to be impacted by TDI *in vitro* Kim et al., (2010); Wang et al., (2017)
Angiogenesis regulation and/or vascular morphogenesis	PTGS1 (aka COX-1)		ETV1, PDZRN3, SALL1			
Lipid metabolism	TNF-α			LEPR		

### Meta-analysis

**FIGURE 3:**
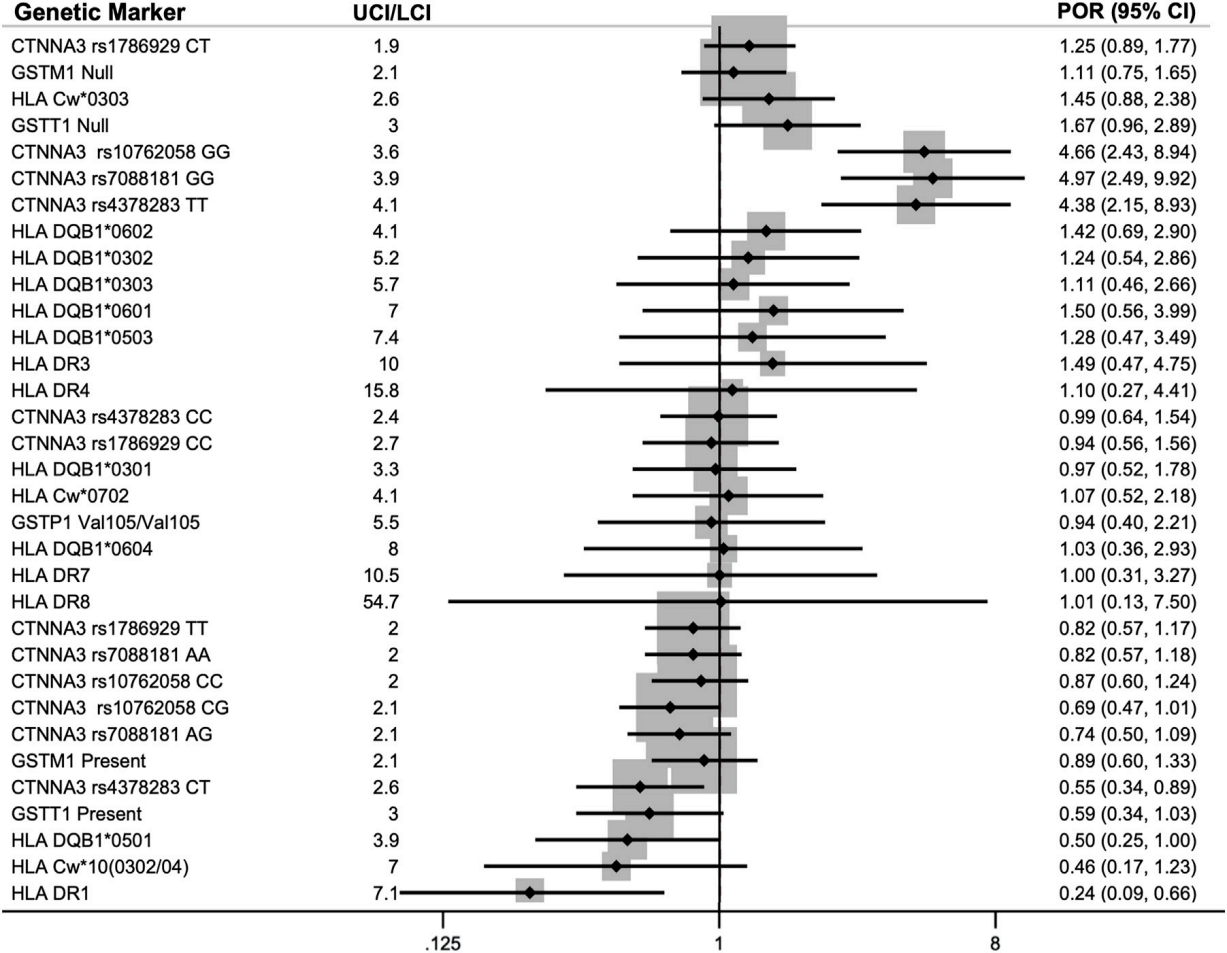
Pooled prevalence odds ratios (POR) for association of genetic markers with diisocyanate asthma (DA) using an inverse-variance model (IV). Each of the 23 genetic markers was reported by two to three studies. The lower 95% confidence interval (LCI) and upper 95% confidence interval (UCI) are provided in parenthesis next to the POR. The meta-analyses included data for asymptomatic worker (AW) controls when available or normal controls (NC) when AWs were not studied. Markers with POR >1.0 indicates a higher prevalence of that genotype within workers with DA (DA risk marker) and markers with POR <1.0 had a lower prevalence within DA workers compared to the control group (protective marker against DA).

The meta-analyses included 23 genetic markers within 11 genes/serotypes: CTNNA3, GSTM1, GSTP1, GSTT1, HLA-Cw, HLA-DQB1, HLA-DR1, HLA-DR3, HLA-DR4, HLA-DR7, and HLA-DR8 (see Figure 3 for the meta-analysis PORs). Fixed effects and random effects PORs were similar to each other (see Supplementary Excel File), and tau-squared estimates of heterogeneity were zero for eleven of the fifteen markers. The precision of the estimates was evaluated using the width of the confidence intervals, in which the UCI was divided by the LCI for the pooled POR, which showed an average of 5 ± 2 (range: 2–8).

### Sensitivity analysis

Sensitivity analysis was performed to evaluate the impact of our decision to use unadjusted PORs and to prioritize using AW controls followed by using NC controls and not using DA-control data in the meta-analysis (see Supplementary Excel File). The sensitivity analysis showed that adjustment for confounders impacted the results more than the choice of the control group did; when authors adjusted for potential confounders it caused the pooled PORs to be closer to the null (i.e., POR closer to 1).

The sensitivity analysis for the 33 genotypes for 23 genetic markers involved analyzing the data seven different ways described in the methods section and then evaluating how consistent the findings were across the analyses. Three genes or serotypes had markers with stronger evidence of their association with DA that was mostly consistent across half or more of the sensitivity analyses: HLA DR1, CTNNA3, and GSTT1 present/null. Genes or serotypes with evidence that had a directional POR in only one or two of the seven analyses and therefore were more sensitive to analysis parameters (i.e., control groups used and adjustment or no-adjustment for potential confounders) were HLA-C, HLA-DQB1, and GSTM1. Genes or serotypes with consistently very little evidence for their association with DA prevalence were GSTP1 Val105/Val105, HLA-DR3, HLA-DR4, HLA-DR7, and HLA-DR8, which had PORs that straddled one (in which a POR of 1 indicates no difference between cases and controls) and were consistently negative across all seven ways of analyzing the data for their association with DA. For the four CTNNA3 SNPs that we were able to analyze using data from the 1,000 genomes project (list 8 in Supplementary File), the results were very similar to the primary meta-analysis using AW and NC control groups (see Supplementary File).

**FIGURE 4:**
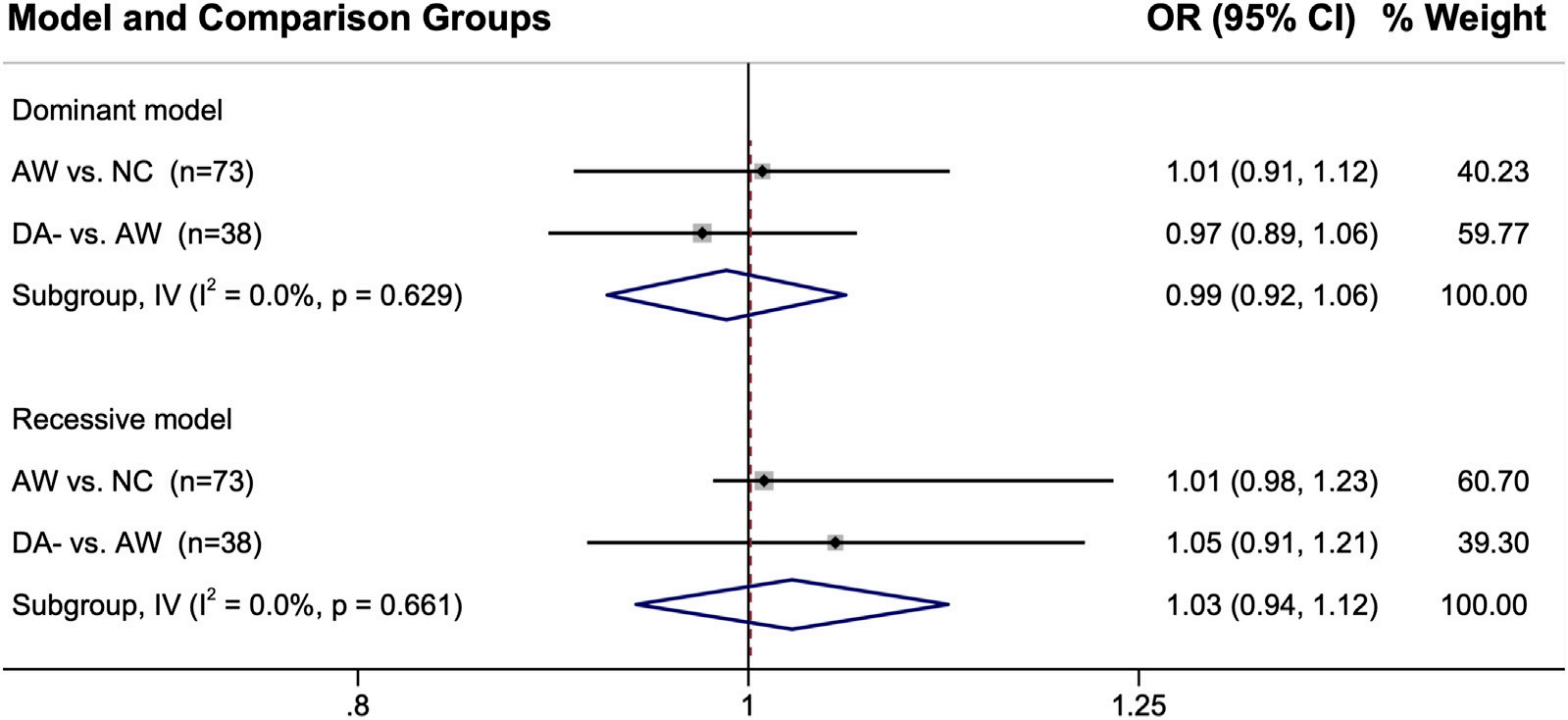
Comparison of the genotype distributions between control groups from the studies in which two control groups were used. When single nucleotide polymorphisms were reported, either the dominant model or the recessive model was used for that particular genetic marker. Abbreviations: AW, asymptomatic exposed worker; 95% CI, 95% confidence interval; DA-, diisocyanate asthma negative worker with respiratory symptoms; I^2^, heterogeneity statistic; IV, inverse-variance statistical model; n, number of genetic marker comparisons; NC, normal non-exposed healthy control; OR, odds ratio.

The genetic marker data for the control groups were also compared directly to each other when more than one group was used for a single study*.* The pooled ORs were all near 1 for these analyses, indicating there are likely negligible differences in their genotype distributions with the ORs showing 1% ⁠— 5% between the control groups (Figure 4).

## Discussion

### Systematic literature review-gene functions

We performed a systematic literature review and meta-analysis in order to evaluate the peer-reviewed published findings about the relationship between genetics and diisocyanate-induced occupational asthma. Some of the most studied genes in the candidate-gene studies were human leukocyte antigen (HLA) serotypes (involved in immune system response), as well as glutathione-S-transferase (GST) and N-acetyltransferase (NAT) genes (both involved in metabolism) (Bignon et al., 1994; Balboni et al., 1996; Bernstein et al., 1997; Mapp et al., 2000; Piirilä et al., 2001; Mapp et al., 2002; Wikman et al., 2002; Beghé et al., 2004; Kim et al., 2006; Choi et al., 2009; Yucesoy et al., 2012; Yucesoy et al. 2014; Yucesoy et al. 2015a). Three exploratory genome-wide association studies (GWAS) indicated additional isocyanate-asthma associations with genes that are involved in cell-cell adhesions and inflammation, including: ATF3 (transcription factor for cellular stress response and lung inflammation and cell-cell adhesions), CDH17 (calcium-dependent membrane-associated glycoprotein for cell adhesions), CTNNA3 (E-cadherin-mediated cell-cell adhesions), HERC2 (protein ubiquitination, antigen processing, and DNA damage response), ODZ3 (homophilic cellular adhesions and regulation of neuronal development), and TACR1 (neurokinin receptor associated with airway hyperresponsiveness, airway inflammation, and asthma) (Kim et al., 2009; Yucesoy et al., 2015a; Bernstein et al., 2018). The GeneMANIA output for these DA-associated genes identified via the systematic literature review shows the overlap in protein network functions for genes associated with isocyanate asthma prevalence when DA cases were compared to controls (AW or NC). The protein functions for these genes included antigen processing (HLA-A, HLA-B, HLA-C, HLA-DPB1, HLA-DQB1, HLA-DRB1, HLA-DQA1), xenobiotic metabolic process (GSTM1, GSTT1, GSTP1, GSTM3, NAT1, NAT2), regulation of apoptosis (TNF, GSTP1), and positive regulation of cytokine production (TNF, HLA-A) (Figure 2).

These functions are involved in immune system response and could be impacting isocyanate-asthma susceptibility by modifying inter-individual inflammatory responses after exposure to isocyanates. A few examples include xenobiotic metabolism (*GST* and *NAT* gene families), immune system function (*HLA* gene family), regulation of cytokine production (*HLA* gene family, *PTGS2, TGFB1, TNF*), and response to oxidative stress (*GSTP1, MBL2, SOD2*) (see NCBI descriptions in Table 2 and GeneMANIA network functions in Figure 2). Multiple studies reported significant markers in the *GST* and *HLA* gene families, which were meta-analyzed and were indicative of protection against DA. The results suggest that the *GST* and *NAT* gene families may be involved in the metabolism of diisocyanates and that *HLA* class I and/or class II genes might contribute to the mechanism of sensitization by diisocyanates. A proposed metabolic pathway for HDI showing the potential involvement of GST and NAT enzymes leading to the formation of HDI-protein adducts and potential immune response and elimination has been previously published (Flack et al., 2010). Additional evidence for the role of GST in the metabolism of diisocyanates includes a study that found administration of glutathione to be protective against diisocyanate toxicity (Wisnewski et al., 2005). HLA class I and class II alleles were studied as candidate genes because of their immune system functions, including processing and presentation of exogenous antigens, which is why they are thought to potentially play an important role in DA development.

The first candidate gene study for GST was conducted by Piirilä and colleagues in 2001 with the hypothesis that glutathione *S*-transferases (GSTs) might conjugate diisocyanates with glutathione and they observed that GSTM1(null) increased the risk of DA (POR = 1.89, 95% CI 1.01–3.52) in the Finnish population (Piirilä et al., 2001). More recently, Yucesoy and colleagues also studied GSTM1(null) but observed a protective effect in a French-Canadian population when combined with other markers in a logistic regression analysis (Yucesoy et al., 2016). However, when they analyzed GSTM1(null) as a stand-alone genotype, the OR was to the right of the null for DA *vs.* DA- (POR 1.1, 95% CI: 0.6–1.9) but was to the left of the null for DA *vs.* AW (POR 0.8, 95% CI: 0.5–1.3) (Yucesoy et al., 2012). This seems to indicate that it is too early to ascertain what the impact is for the GSTM1 null genotype on the risk for a worker to develop DA, thus more research is needed.

It is also important to note that the GeneMANIA networks might include false positive genes that, with additional studies, may prove not to be associated with DA outcome. For example, it is not possible at this time to determine whether associated SNPs are the causative marker or if there are proximal functional SNPs in linkage disequilibrium with the correlated SNPs. A more in-depth discussion of the potential biological relevance of the *GST* and *HLA* genes can be found in the original publications (Bignon et al., 1994; Piirilä et al., 2001; Mapp et al., 2002; Choi et al., 2009; Yucesoy et al., 2012) and in a review by Bernstein and colleagues on the genetics of DA (Bernstein 2011).

### Meta-analysis on genetic markers associated with DA

There were 11 genes or serotypes with 23 markers that were reported by two or three groups and thus conformed to meta-analysis. The genes and serotypes with markers that were meta-analyzed were: *CTNNA3*, *GSTM1, GSTP1, GSTT1, HLA-C, HLA-DQB1*, *HLA-DR1, HLA-DR3, HLA-DR4, HLA-DR7, and HLA-DR8* (Bignon et al., 1994; Bernstein et al., 1997; Mapp et al., 2000; Piirilä et al., 2001; Mapp et al., 2002; Beghé et al., 2004; Kim et al., 2006; Choi et al., 2009; Kim et al., 2009; Yucesoy et al., 2012; Bernstein et al., 2013). Most of the meta-analyzed pooled PORs and CIs straddled the null hypothesis of no effect. Currently, the strongest evidence of association with DA prevalence was for CTNNA3 and HLA-DR1.

The strongest POR effect sizes were for catenin alpha 3 (CTNNA3) SNPs and they had more precise confidence intervals than most of the other genetic markers studied. CTNNA3 is a gene that codes for a protein that is important for stretch-resistant cell-cell adhesions and was studied by two groups (Kim et al., 2009; Bernstein et al., 2013). Initially, three CTNNA3 SNPs were found to be significantly correlated with increased DA risk in a Korean GWAS by Kim and colleagues in 2009 (rs10762058 GG, rs7088181 GG, rs4378283 TT) compared to normal non-exposed controls (Kim et al., 2009). A few years later, Bernstein et al. replicated these results in a Caucasian French-Canadian and Spaniard population for two of those three SNPs, rs10762058 and rs7088181, when comparing DA workers to exposed asymptomatic workers (noting that no association was found when compared with symptomatic DA-workers as the control group) (Bernstein et al., 2013).

When the NC and AW results from Kim and Bernstein’s studies (Kim et al., 2009; Bernstein et al., 2013) were meta-analyzed, all three CTNNA3 SNPs showed the odds of DA cases having those genotypes (rs10762058 GG, rs7088181 GG, rs4378283 TT) was between four to five times greater than controls without DA (PORs 4.38–4.97). The hypothesis for this association was that differences in cell-cell adhesions may result in increased permeability of diisocyanates in lung tissues (Bernstein et al., 2013), resulting in higher levels of absorption into the body when workers are exposed to diisocyanates. Soon afterward, another group investigated the association of CTNNA3 with asthma and confirmed that CTNNA3 is expressed in the lung and their knock-out mouse model was hyperresponsive to methacholine (Folmsbee et al., 2015). The ability of Bernstein et al. (2013) to replicate the GWAS results with exposed healthy controls in a separate ethnic population from Kim et al. (2009) combined with the animal research provides compelling evidence of the involvement of CTNNA3 and the importance of cell-cell adhesions in DA etiology.

The other genetic marker that showed association with DA prevalence after meta-analysis was HLA-DR1 serotype, which was protective against DA with a pooled POR of 0.24. By taking the inverse of the POR, this means that workers without HLA-DR1 serotype were more than four times more likely to have DA than controls. HLA-DR1 is one of more than 16 specificities of HLA-DRB1 (Moreland 2004) and was reported on by three groups: Bignon et al., 1994, Bernstein et al., 1997, and Mapp et al., 2000. HLA-DR1 is involved in T-cell response and has also been associated with decreased risk of other immune system diseases including rheumatoid arthritis (Rosloniec et al., 2002) and multiple sclerosis (Luomala et al., 2001).

It is notable, however, that only 2 or 3 studies reported findings on each of these genetic markers for meta-analysis. Additionally, the confidence intervals for the meta-analyzed markers spanned a substantial range (the average UCL/LCI was 6 ± 3), demonstrating that there is still a lot of uncertainty about the true PORs for these markers. Thus, more research needs to be conducted in order to better understand the potential role of these genetic markers in affecting DA prevalence.

### Bioinformatics

Predicted protein network functions for the genetic markers associated with prevalence of DA showed a lot of overlap with diisocyanate research investigating genes associated with variation in biomarker levels before asthma develops. Bioinformatics analysis revealed that inflammatory pathways appear to be important in both diisocyanate biomarker and asthma research. Protein pathways for genetic and epigenetic markers that are associated with differences in biomarker levels and/or asthma risk have functions including but not limited to: immune function (HERC2, HLA serotypes), cell-adhesions (ATF3, CDH17, CTNNA3, KRT6A, ODZ3), metabolism (NAT and GST gene families), and inflammation (ATF3, TACR1, TGF-β) (see Table 3). Regulation of cell migration, calcium levels, transcription, and neurotransmitters were also recurrent themes. The findings suggest that protein pathways that impact variability in worker biomarker levels can also impact asthma susceptibility. This could potentially occur if toxicodynamic effects like inflammation, which results in blood vessels becoming more permeable, impact isocyanate toxicokinetics in chronically exposed workers in a way that increase the absorption of diisocyanates into the body, delay their excretion, and thereby increase the risk of immune system sensitization and asthma development over time.

Analyzing the predicted protein pathways for overlap in published findings demonstrates that diisocyanate research findings have greater reproducibility than would be concluded if only the specific genetic markers were examined for overlap. By focusing on predicted protein pathway functions, overlap is observed across *in vitro, in vivo,* human studies, genetic studies, epigenetic studies, asthma research, and biomarker research for diisocyanates (see Table 3). Focusing on these protein functions can help to generate new hypothesis-driven research into the mechanisms by which diisocyanates cause respiratory sensitization and by extension, research into how to prevent DA from occurring.

### Bias assessment

All of the included studies were case-control candidate gene or GWAS designs. While case-control studies have many benefits (e.g., retrospective with no long follow-up period, cost-effective, efficient for rare outcomes), they are also prone to several forms of bias that can impact the results. Some of the biases that could be impacting the findings of the included studies are publication bias, selection bias, and ascertainment bias. These are our main validity concerns, but the small numbers of published results per genetic variable (in part because of the publication bias) prevented us from conducting the stratified and meta-regression analyses of validity characteristics that we would ordinarily conduct in a review of a more fully reported literature.

There was substantial publication bias from researchers who only published their statistically significant findings and did not report the data for all of the insignificant markers that were studied too. We contacted all of the research groups for missing information but none of them were able to provide us with additional data or other pertinent information. Unreported data is a very concerning problem that could result in researchers placing too much focus on genetic markers that appear to have an association with DA. For example, if one study found a significant DA association but a majority of studies observed that the genetic marker is insignificant, then it could indicate that the marker is not actually of interest when the results are meta-analyzed. Instead, the genetic marker may have been a false positive result in the one study. This cannot be determined though if the statistically insignificant results are never published. Thus, it is critical for researchers to publish all of their findings, including by uploading the full results data into a public repository when performing a GWAS.

Selection bias can occur when the included subjects are unrepresentative of the population from which the cases arise. This is a problem for the NC group who were unexposed and are therefore not representative of the population from which DA cases arise. Furthermore, unfortunately, the extent to which selection bias might be influencing the current body of research for the DA- and AW comparisons is not possible to evaluate because not enough information was provided about the recruitment of the cases and controls. Sometimes information about which industry the workers were in when they were exposed to diisocyanates and/or which clinic the cases were recruited from was reported, but often no other recruitment details were included. Because of this, selection bias cannot be sufficiently compared between studies. For example, volunteer bias could not be compared across studies because no details were provided about how cases and controls were contacted about recruitment for the studies (such as by using fliers, making phone calls, etc.). Most researchers also did not report whether the cases and DA- or AW controls were matched in any way, such as by recruiting controls from the same workplaces where the cases had worked. If controls were recruited from different workplaces, communities, industries, countries, etc. then they may not be a representative control group for the worker populations from which the cases arose.

There may also be some ascertainment bias of the cases and controls if there were false-positive DA or false-negative DA classifications of workers. False-positive DA classifications are rather unlikely, but false negatives may be of concern because DA can take up to ten or more years to develop (Malo et al., 1992). This means that the subjects from the control groups all have the potential to later develop DA. Thus, there is likely some ascertainment bias impacting results (particularly for DA- and NC control groups).

### Limitations

The main limitations of this meta-analysis are (1) that there were few studies that investigated overlapping genetic markers and (2) that the published literature is profoundly affected by publication bias from insignificant results not consistently being reported. Additionally, the industrial use or occupational exposure settings (which would help predict the main exposure route being through skin or inhalation) were often not reported and the studies either included only TDI-exposed workers or a mix of workers who were exposed to HDI, MDI, TDI, or a combination of diisocyanates. Thus, we were unable to analyze whether the type of diisocyanate or the main exposure route influence which genes are associated with DA. Another limitation is that only a limited number of research groups have studied genetic associations with DA and there was limited diversity across the populations that were studied.

As such, this work should be used for generating new hypothesis-driven research and to better understand the predicted protein functions that may be impacting DA etiology. More studies are required to gather further evidence for whether any of these specific genetic markers increase or decrease worker risk of developing DA. Additionally, Table 3 should not be considered to be fully comprehensive as it is a compilation of several trends we noticed but it was not compiled systematically like the rest of the work.

### Suggestions for future research

When selecting a control group, it is important to consider the pros and cons of each choice. For our main analysis we used data for AW when available, or NC when AWs were not studied [ranked list of preference: AW, NC (no DA-)]. AW controls in the included studies had more than 10 years of exposure to diisocyanates on average and had remained asymptomatic, making them the most reliable true negative controls who are the least likely to later develop DA. On the other hand, we believe that some of the DA-controls may have had false negative results for their medical testing because those workers had already developed respiratory symptoms and might actually belong in the DA group (especially if they have a delayed response to diisocyanate SIC testing). It is also possible that DA-workers are more likely to develop DA later if they continued to be exposed to diisocyanates; this could particularly pertain to the workers who had less than 10 years of occupational exposure. Sometimes DA takes many years to develop; Malo and colleagues reported that 40% of workers in their study developed DA after more than 5 years of work (Malo et al., 1992). Additionally, for the NC group, some people who have a genetic susceptibility for becoming a DA case would have been classified as a negative control even though they may have become a DA case if occupationally exposed to diisocyanates, but much more precision is gained through using NC to greatly increase the size of the control group.

Encouragingly, the comparisons of the control groups directly against each other shown in Figure 4 provides evidence that the genetic differences between the different types of control groups were minor, with the point estimates within 1–5% of the null hypothesis of OR = 1. However, some of the confidence intervals were wide (and was right-shifted when using the recessive genotype model for AW *vs.* NC). Additionally, there were only a few genes for which this direct comparison between the control group genotypes could be performed (38 genes for DA- *vs.* AW and 73 genes for AW *vs.* NC; see Supplementary Excel File). Furthermore, DA- *vs.* NC could not be compared because those two groups were never reported in the same study. Thus, further analyses would be useful for better understanding the differences and similarities between the DA-, AW, and NC control group genetic profiles. These initial results are encouraging though about the overall similarity in the genetics of the different control groups.

In future research on the impact of genetics on DA prevalence, it would be beneficial for investigators to study genetic markers that overlap with previous research. Even though many of the genetic markers that were reported in the literature were often within the same genes as reported in the previous research (such as the *HLA* and *GST* gene families), the specific markers that were studied (e.g., SNPs, serotypes) were almost always different and therefore most of the findings could not be meta-analyzed. Over eighty genes/serotypes and nearly one thousand POR assessments of specific markers were reported in studies on DA but at most only two or three publications had data reported for the same markers. Also important to recognize is that more markers have been tested than that, but the GWAS studies did not report results for all of the SNPs they tested, and sometimes they only reported p-values and not a POR for SNPs. However, PORs are needed to better understand the effect size of the genotype on the prevalence of DA. As a result, only 33 POR assessments for markers within 11 genes/serotypes could be meta-analyzed, which is only 3.5% of all reported data for 943 genetic marker POR comparisons. The lack of overlap for meta-analyzing results across research groups could become more infrequent as GWAS replace candidate gene studies, but only if the researchers report POR findings for all SNPs in the GWAS (i.e., both the significant and insignificant comparisons).

The sensitivity analysis showed that author-adjustment for confounders had little impact overall and primarily diluted the findings of the genetic associations by moving the POR estimates closer to the null in most cases. When deciding whether to perform logistic regression with adjustment for potential confounders, additional analysis should be done to determine whether a variable is truly a confounder or not. Age, sex, smoking, and atopy were the most common variables that were included as covariates, but these may not be appropriate variables to include in these models. Some groups reported in their methods that they tested the variables in logistic regressions (Bernstein et al., 2013). It is unclear though how it was decided whether the variable was a covariate (presumably it may have been decided by checking whether the p-value for the variable was less than 0.05 in the model). Confounders should be variables (or close proxies) that cause the exposure variable being studied and/or the outcome (i.e., DA in this case) (VanderWeele. 2019). Before including variables in a model, methods such as creating directional acyclical graphs (DAGs) and/or using multidimensional scaling (MDS) methods can help elucidate whether or not potential covariates are actual confounders for the study population in question. Researchers should be careful when selecting covariates to add to a statistical model because including variables that are not confounders can introduce and/or amplify bias in the analysis (VanderWeele et al., 2019). An in-depth discussion of selecting covariates using DAGs is provided by VanderWeele and colleagues (VanderWeele. 2019).

The lack of reporting results for all of the genetic markers that were studied is a problem that must be addressed in future studies. The currently published GWAS and some of the candidate-gene studies only reported statistically significant findings, which causes publication bias and reduces the number of markers that can be assessed with meta-analysis to better estimate the true effects and evaluate the consistency of findings. In addition to not always reporting all insignificant results, there were often other important details that were left unreported. Every publication reviewed had missing information. Some examples of information that should have been reported by the research groups include: the HWE p-value cut-off used for controls, whether the cases and controls were matched in any way (for example, by workplace location), the duration of diisocyanate exposure, the industry the workers worked in, the type of diisocyanate to which the workers were exposed, whether any of the cases or controls overlapped with another published study, how workers were recruited, and what the non-response rates were for cases and controls. Researchers should be careful to include more information about the history and recruitment of cases and controls when they publish studies in the future and must begin to report data for all of their findings (all significant and insignificant genetic markers).

## Conclusion

This systematic literature review presents protein network functions for genes that have had markers reported to be associated with DA, and the meta-analyses provide new insights into which genetic markers have the most compelling evidence within the present literature. Specifically, genetic markers within the *GST, CTNNA3*, and *HLA* gene families were protective in the meta-analysis, suggesting that *GST* genes might be involved in the metabolism of diisocyanates, *CTNNA3* may impact tissue permeability and affect exposure uptake, and the *HLA* gene family might influence immune system response in a way that alters worker risk of developing DA. More research needs to be conducted to better understand the relationship between various genetic markers and DA risk.

In this review, we found that the main challenge for meta-analysis is that the literature is profoundly publication biased. We discuss how study design and reporting can be improved in future studies on the genetics of DA, or any other disease with a genetic component. When deciding which genetic markers to study in future candidate gene studies, researchers can refer to the Supplementary Excel File to identify genetic markers (SNPs, serotypes, haplotypes) that have been investigated by other groups, which would increase the utility of future meta-analyses. Studying markers within the same genes is not enough; the same markers must be investigated in multiple studies for meta-analysis of the results to be able to ascertain whether previous research findings are reproducible and to better understand the magnitude of effect. When performing logistic regressions to control for potential confounders, the variables should be assessed (such as with DAGs and/or MDS methods) for each study population instead of being included as covariates by default. Finally, researchers need to report more information about the history and recruitment of the workers and they must report the prevalence odds ratio results for every genetic marker that they study regardless of statistical significance (for GWAS, results should be uploaded to data repositories), which would reduce publication bias from impacting future meta-analyses.

## Data Availability

The original contributions presented in the study are included in the article/Supplementary Material, further inquiries can be directed to the corresponding author.
